# Dynamic nomogram for predicting early tracheotomy in patients diagnosed with supratentorial deep seated intracranial hemorrhage

**DOI:** 10.3389/fneur.2025.1670672

**Published:** 2025-11-05

**Authors:** Chubin Liu, Suqiong Yang, Gang Wang, Jiayin Wang, Liangqin Luo, Yasong Li

**Affiliations:** ^1^Department of Neurosurgery, Second Affiliated Hospital of Fujian Medical University, Quanzhou, China; ^2^Department of Psychiatry, The No. 3 Hospital of Jinjiang City, Jinjiang, China; ^3^Department of Neurosurgery, The Second Hospital & Clinical, Medical School, Lanzhou University, Lanzhou, China

**Keywords:** early tracheotomy, supratentorial deep-seated intracranial hemorrhage, dynamic nomogram, predicted model, clinical application

## Abstract

**Background:**

Tracheotomy (TT) is frequently performed in patients diagnosed with supratentorial deep-seated intracranial hemorrhage (SDICH). However, predicting whether early TT is necessary remains a challenge for neurosurgeons. As such, the present study constructed a dynamic nomogram prediction algorithm to determine whether patients with SDICH immediately required early TT on arrival to hospital.

**Methods:**

Clinical and baseline data from patients diagnosed with SDICH at The Second Affiliated Hospital of Fujian Medical University (Fujian, China) and The Second Hospital & Clinical Medical School of Lanzhou University (Gansu, China) between January 1, 2019 and January 1, 2023 were retrospectively collected and analyzed. A dynamic nomogram prediction model was constructed and used to examine the impact on early TT endpoints.

**Results:**

Data from 1,046 patients with SDICH fulfilled the inclusion and exclusion criteria. Of these, 379 patients from Lanzhou University Second Hospital comprised the external validation set and 667 from The Second Affiliated Hospital of Fujian Medical University comprised the training set. A total of 199 (19.02%) patients underwent early TT. White blood cell (WBC), platelet (PLT), heart rate (HR), and Glasgow Coma Score (GCS) were used to build a dynamic nomogram prediction model. An area under the curve (AUC) of receiver operating characteristic (ROC) was 0.817, and 95% confidence interval (CI) of 0.785–0.845 were obtained from ROC curve analysis of data from the training set, cut-off value of training set was >0.139. The AUC was 0.768 in the validation set (95% CI 0.722–0.809), and cut-off value was >0.182. A strong association was found between observation and prediction of early TT according to dynamic nomogram calibration curves and clinical decision curve analysis.

**Conclusion:**

A dynamic nomogram prediction model for early TT in patients diagnosed with SDICH was developed and validated. GCS, WBC, PLT, and HR were valid markers for early requirement of TT.

## Introduction

Supratentorial deep-seated intracranial hemorrhage (SDICH) is a severe cerebrovascular condition associated with significant morbidity and mortality (1). The affected regions, including the thalamus and basal ganglia, are critical for neurological functions, and hemorrhage in these areas often results in poor clinical outcomes (2). Respiratory complications, particularly prolonged mechanical ventilation, are common in patients with SDICH due to reduced consciousness and impaired airway protection (3, 4). Tracheotomy (TT) is a well-established neurocritical care procedure that secures the airway, reduces complications of prolonged intubation, and facilitates ventilation. Early TT, performed within 7 days of admission, has been shown to shorten the duration of mechanical ventilation and intensive care unit (ICU) stays, potentially improving neurological outcomes (5–7). However, the decision to perform early TT remains challenging because of patient variability and the lack of reliable predictive tools to guide timely intervention.

Recent studies have developed scoring systems such as SET and TRACH to predict the need for TT (8–10). However, these models are either too complex for urgent clinical use or do not specifically address early TT in patients with SDICH. The application of machine learning and dynamic prediction models in clinical decision-making has emerged as a promising approach to improve prediction accuracy for early TT in patients with SDICH in neurocritical care (11–13). These models provide real-time individualized risk assessments and can be integrated into clinical workflows, offering advantages over static scoring systems.

In this study, we developed and validated a dynamic predictive nomogram to assess the need for early TT in patients with SDICH. Our model incorporated readily available clinical parameters, including Glasgow Coma Scale (GCS), white blood cell (WBC) count, platelet (PLT) count, and heart rate (HR), which have been identified in previous studies as significant predictors of airway complications in patients with severe brain injury. Unlike previous models, our dynamic nomogram is accessible through a web-based platform, allowing rapid clinical decision-making at the point of care. The model was rigorously validated using an external cohort, further supporting its reliability and generalizability.

This tool provides clinicians with a practical and effective method to predict the need for early TT. Dynamic nomograms have notable advantages over existing predictive models because they are user-friendly and adaptable to individual patient profiles, thereby meeting the growing demand for precision medicine in neurocritical care.

## Materials and methods

### Study population

This retrospective observational study was conducted at two medical centers. To rigorously validate the generalizability of the model, the cohort was divided into training and external validation sets based on the source hospital. The training set consisted of consecutive eligible patients from The Second Affiliated Hospital of Fujian Medical University, while the external validation set included consecutive eligible patients from Lanzhou University Second Hospital. This allocation ensured strict geographical and administrative separation, with no temporal or patient overlap between the sets, thereby providing a robust test of external validity. A total of 1,394 patients diagnosed with SDICH between January 1, 2019, and January 1, 2023, were identified. SDICH was defined as a spontaneous hemorrhage located in deep cerebral structures, including the basal ganglia and thalamus, confirmed by computed tomography (CT). After applying the inclusion and exclusion criteria, 1,046 patients with CT-confirmed SDICH were enrolled. Of these, 667 patients from Fujian Medical University Second Affiliated Hospital comprised the training set, and 379 patients from Lanzhou University Second Hospital comprised the validation set.

**Figure 1:**
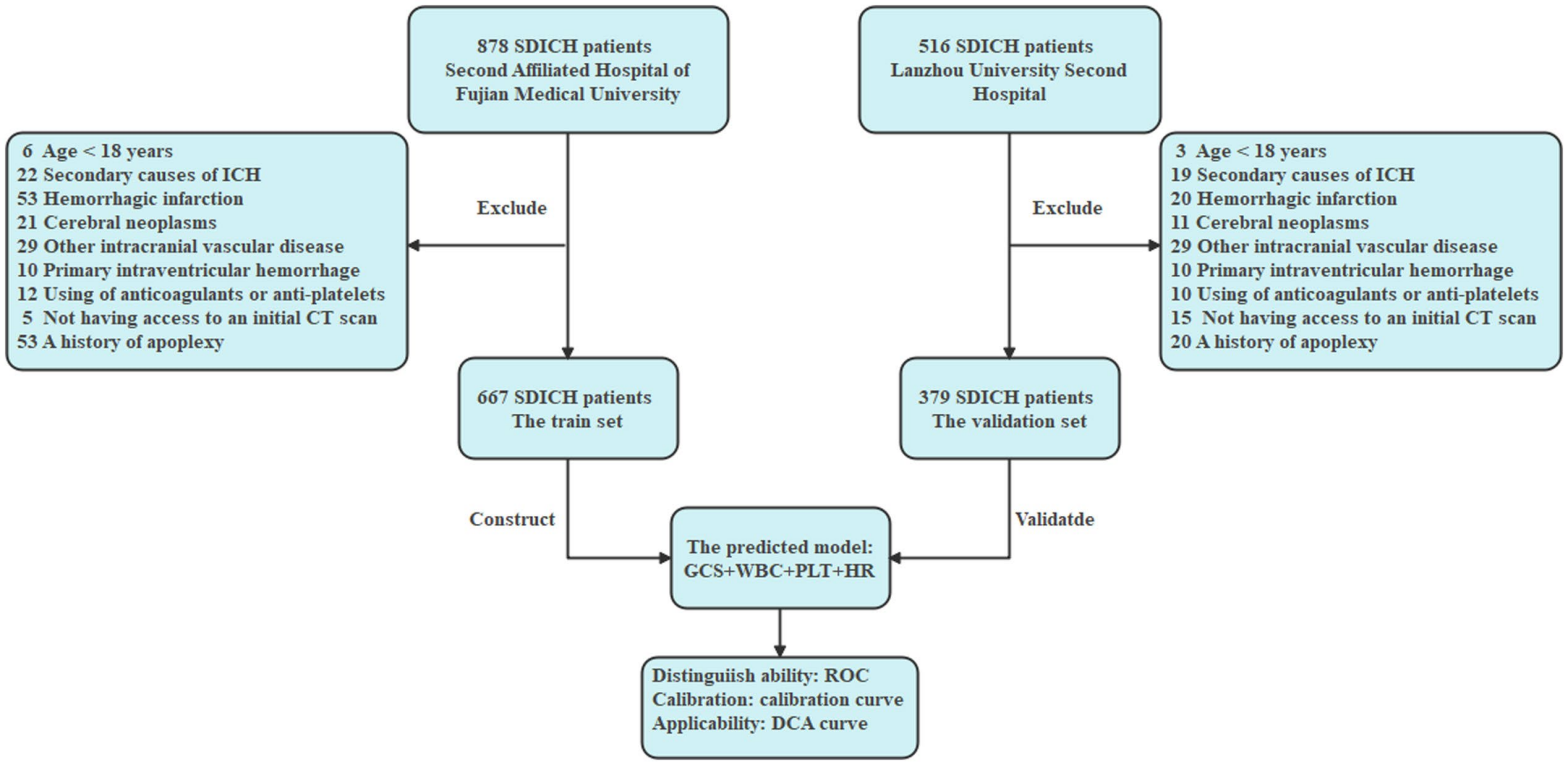
Flowchart.

The inclusion criteria were: (1) CT-confirmed diagnosis of SDICH, as defined above; (2) Hospital stay sufficient duration to determine early TT status (i.e., ≥7 days or discharge/death within 7 days); (3) Age ≥18 years. One patient fulfilled the following specific criteria for exclusion: (1) age <18 years; (2) secondary causes of intracranial hemorrhage (ICH); (3) hemorrhagic infarction, cerebral neoplasms, intracranial aneurysm, arteriovenous malformation, or moyamoya disease; (4) primary intraventricular hemorrhage; (5) ICH related to anticoagulant or antiplatelet use; (6) absence of an initial CT scan; (7) history of apoplexy (see Figure 1).

### Ethical statement

This retrospective study was conducted in accordance with the Declaration of Helsinki and was approved by the Ethics Committees of The Second Affiliated Hospital of Fujian Medical University [(2023) The Second Affiliated Hospital of Fujian Medical University Ethical Review (677)] and The Second Hospital & Clinical Medical School of Lanzhou University (The Second Hospital & Clinical, Medical School, Lanzhou University Ethical Review: 2020A-253). The need for individual informed consent was waived by both ethics committees due to the retrospective nature of the study.

### Define early TT and the outcome

The primary outcome was “early TT,” defined as a tracheotomy procedure performed within the first 7 days after hospital admission. Patients who did not undergo tracheotomy, or who underwent it after day 7, constituted the Non-TT group.

### Baseline data collection

Patient demographic and clinical data, including age, sex, diabetes, and hypertension history, were carefully recorded. The interval between symptom onset and the first CT scan, along with laboratory results obtained at admission, was documented. Data were extracted from a state-of-the-art electronic health record system. On admission, on-call neurosurgeons thoroughly evaluated GCS scores. ICH volume was calculated using the Tada formula, which applies the ABC/2 method to the original CT scan, incorporating radius values in all three dimensions (Table 1).

**Table 1:** Baseline characteristics of training set and validation set.

	Training set			Validation set		
Characteristics	Non-TT	TT	*p*-value	Non-TT	TT	*p*-value
	(*N* = 580)	(*N* = 87)		(*N* = 267)	(*N* = 112)	
Age median (IQR)	57.5 (52.0–66.0)	56.0 (50.0–66.0)	0.363	58.0 (50.0–67.5)	56.00 (49.0–68.0)	0.470
Gender (*N*, %)			0.906			0.135
Male	362 (62.4)	59 (68.2)		153 (57.3)	74 (66.1)	
Female	218 (37.6)	28 (31.8)		114 (42.7)	38 (33.9)	
Medical history						
HP (*N*, %)	371 (64.5)	59 (68.2)	0.481	176 (65.78)	76 (68.2)	0.001
DM (*N*, %)	66 (11.4)	17 (19.5)	0.048	33 (12.4)	17 (15.2)	0.566
Vital signs median (IQR)						
T	36.75 (36.50, 37.10)	36.90 (36.55, 37.2)	0.030	36.60 (36.50, 36.80)	36.50 (36.50, 36.70)	0.150
HR	80.00 (72.00, 90.00)	86.00 (74.50, 99.0)	0.005	80.00 (74.00, 89.00)	80.00 (75.00, 90.00)	0.224
SBP	155.00 (145.00–165.00)	162.00 (150.50–176.50)	<0.001	165.00 (147.5, 187.00)	181.00 (153.75–196.00)	0.001
DBP	88.00 (78.00–96.25)	90.00 (82.00–100.00)	0.023	91.00 (80.00–102.50)	94.50 (85.00–105.00)	0.259
GCS	12.00 (9.00–14.00)	8.00 (7.00–9.50)	<0.001	12.00 (7.00–14.50)	6.00 (5.00–9.00)	<0.001
HV (mL)	12.79 (4.46–27.86)	30.25 (15.36–41.99)	<0.001	26.00 (19.43, 40.00)	50.00 (35.00, 64.86)	<0.001
ML (mm)	0.00 (0.00–0.00)	0.00 (0.00–7.20)	<0.001	3.20 (2.00–5.60)	7.00 (4.30–11.98)	<0.001
Lab test mean ± SD						
WBC (10^9^/L)	9.19 ± 3.26	11.39 ± 3.99	<0.001	10.86 ± 3.96	12.96 ± 4.84	<0.001
Neutrophile (10^9^/L)	7.63 ± 3.29	9.85 ± 4.00	0.030	8.51 ± 4.02	10.53 ± 4.83	<0.001
Monocyte (10^9^/L)	0.40 ± 0.20	0.47 ± 0.23	<0.001	0.57 ± 0.25	0.65 ± 0.30	0.007
Hb (g/L)	149.62 ± 19.52	153.61 ± 21.42	0.080	139.81 ± 18.12	138.29 ± 19.68	0.467
PLT (10^9^/L)	176.68 ± 60.12	161.79 ± 53.54	0.029	238.90 ± 65.70	228.75 ± 72.40	0.184
Glucose (mmol/L)	7.56 ± 2.32	8.48 ± 2.45	0.001	8.31 ± 3.06	9.37 ± 3.29	0.003
UA (μmol/L)	288.94 ± 104.81	304.34 ± 112.12	0.206	315.31 ± 117.22	375.71 ± 117.59	<0.001
K (mmol/L)	3.53 ± 0.42	3.49 ± 0.46	0.509	3.59 ± 0.46	3.44 ± 0.58	0.009
Na (mmol/L)	137.65 ± 3.47	137.69 ± 3.55	0.916	138.61 ± 4.88	139.27 ± 3.19	0.190
Ca (mmol/L)	2.36 ± 0.15	2.37 ± 0.15	0.923	2.24 ± 0.18	2.26 ± 0.15	0.273
P (mmol/L)	0.88 ± 0.23	0.82 ± 0.26	0.041	0.98 ± 0.28	0.93 ± 0.36	0.124
Mg (mmol/L)	0.85 ± 0.09	0.84 ± 0.10	0.140	0.85 ± 0.11	0.84 ± 0.10	0.462
Albumin (mmol/L)	40.96 ± 3.93	41.60 ± 4.11	0.155	39.24 ± 5.15	36.15 ± 5.69	<0.001

SBP, systolic blood pressure; DBP, diastolic blood pressure; T, temperature of body; GCS, Glasgow Coma Score; HV (mL), hemorrhage volume; ML (mm), midline line shift; WBC, white blood cell; Hb, hemoglobin; PLT, platelet; UA, uric acid; K, kalium; Na, natrium; Ca, calcium; P, phosphorus; Mg, magnesium.

Any intervention performed within the first 7 days after admission was defined as early TT. Treatment plans were individualized for each patient according to their specific needs and implemented in line with the protocols of the two hospitals.

### Statistical analysis

All statistical analyses were conducted using R statistical software (version 4.2.2; R Foundation for Statistical Computing). Continuous variables were tested for normality with the Shapiro–Wilk test. Normally distributed data are reported as mean ± standard deviation and compared using the independent samples *t*-test, whereas non-normally distributed data are presented as median (interquartile range) and compared using the Mann–Whitney *U* test. Categorical variables are expressed as counts (percentages) and compared using the chi-square test or Fisher’s exact test, as appropriate. Statistical significance was defined as a two-sided *p*-value <0.05. All clinically relevant variables collected at admission were considered candidate predictors. Variables with *p* < 0.1 in univariate logistic regression were entered into a multivariable logistic regression model using the backward likelihood ratio method, with an elimination threshold of *p* < 0.05, to develop the final prediction model. Linearity of continuous predictors was assessed using restricted cubic splines (Supplementary Table 1 and Supplementary Figure 1), and multicollinearity was evaluated using variance inflation factors (VIF <1.5) (Supplementary Table 2). Internal validation was performed via bootstrap resampling (1,000 iterations) to obtain optimism-corrected performance metrics. Model discrimination was evaluated using the area under the receiver operating characteristic curve (AUC) with a 95% confidence interval (CI). Calibration was assessed visually using calibration plots and statistically using the calibration slope and intercept. The optimal probability cutoff was determined with the Youden index, and clinical utility was evaluated across a range of thresholds using decision curve analysis.

The proportion of missing data for variables in the final model was negligible (<1%); therefore, complete-case analysis was appropriate.

## Results

### Baseline characteristics of the study participants

As shown in Table 1, the study included 667 and 379 patients in the training and external validation sets, respectively. Comparative analysis revealed significant differences between patients who underwent early TT and those who did not (non-TT group). In the training set, the TT group demonstrated more severe neurological impairment, with significantly lower GCS scores (8.00 vs. 12.00, *p* < 0.001) and larger hemorrhage volumes (HV) (30.25 mL vs. 12.79 mL, *p* < 0.001). They also exhibited signs of heightened systemic stress and inflammation, including elevated HR (86.00 vs. 80.00, *p* = 0.005), higher blood pressure, increased WBC (11.39 vs. 9.19 × 10^9^/L, *p* < 0.001), and glucose levels (8.48 vs. 7.56 mmol/L, *p* = 0.001), along with lower PLT counts (161.79 vs. 176.68 × 10^9^/L, *p* = 0.029). Similar trends were observed in the validation set, where the TT group showed lower GCS scores, larger HV, higher WBC and glucose levels, a higher prevalence of hypertension, and notably lower serum albumin levels (36.15 vs. 39.24 mmol/L, *p* < 0.001). Across both cohorts, there were no significant differences in age or sex, underscoring the predictive importance of these clinical parameters for early TT.

### Comparative analysis of baseline characteristics between centers

A comparative analysis of baseline characteristics between the two medical centers revealed significant differences, highlighting the distinct clinical profiles of the training and validation sets. Patients in the validation set presented with greater initial severity, including lower GCS scores (9.73 vs. 10.94, *p* < 0.001), larger HV (35.44 mL vs. 20.82 mL, *p* < 0.001), and greater middle line shift (5.06 mm vs. 1.42 mm, *p* < 0.001) compared with the training set. The validation cohort also showed more pronounced systemic involvement, with higher systolic and diastolic blood pressure (SBP: 168.99 vs. 157.33, *p* < 0.001; DBP: 93.57 vs. 88.72, *p* < 0.001), elevated inflammatory markers such as WBC (11.48 vs. 9.48 × 10^9^/L, *p* < 0.001) and its subtypes, and a higher prevalence of hypertension (38.52% vs. 11.09%, *p* < 0.001). In contrast, patients in the training set had significantly higher hemoglobin (150.14 vs. 139.36 g/L, *p* < 0.001) and albumin levels (41.04 vs. 38.33 mmol/L, *p* < 0.001). These differences highlight the heterogeneity between the cohorts and strengthen the external validation process by testing the model across diverse patient populations.

### Constructing and testing the prediction dynamic nomogram model

**Figure 2:**
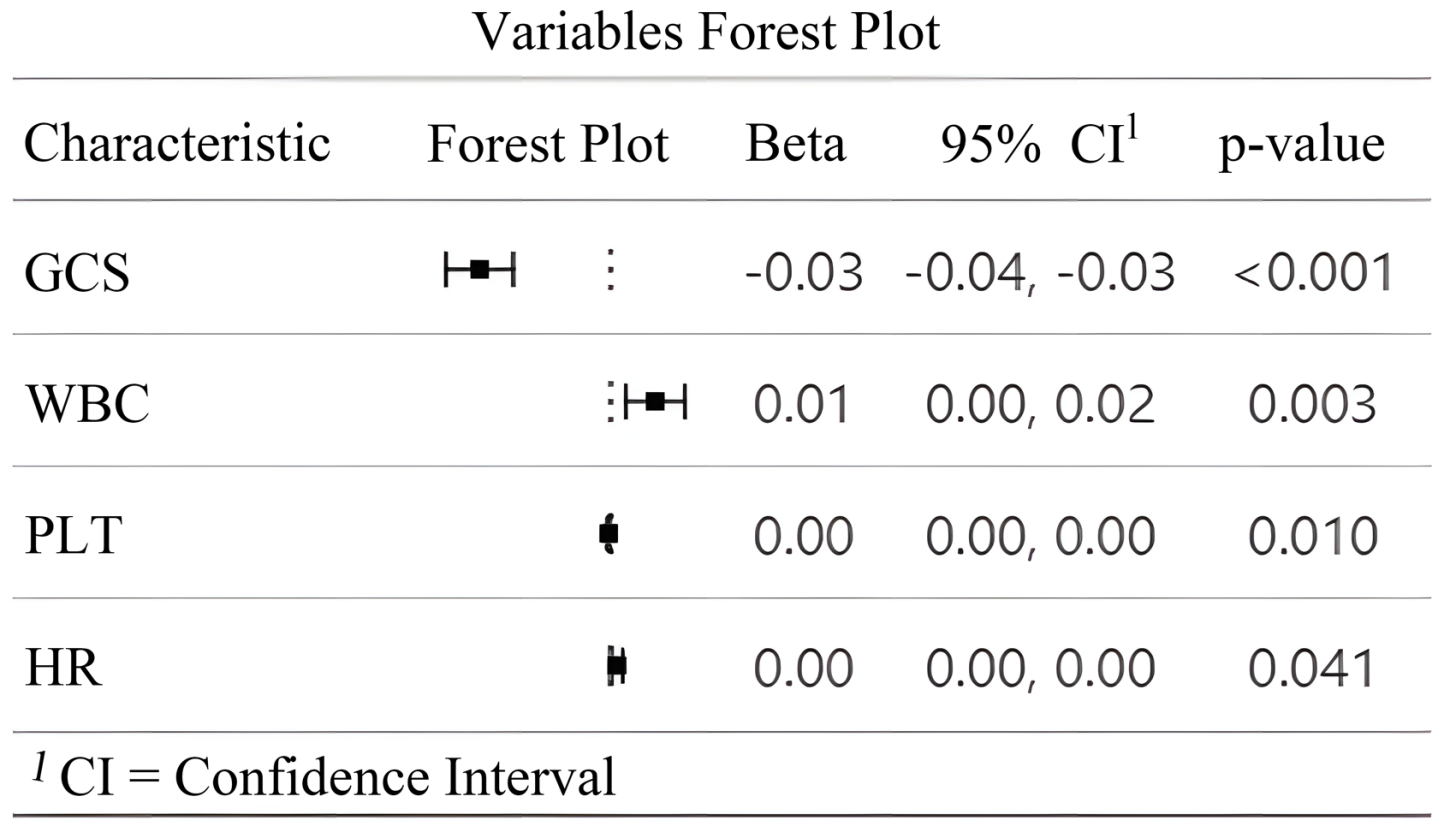
Forest plot of multivariate analysis for predictors of early tracheotomy. This figure illustrates the results of the multivariate logistic regression analysis identifying independent predictors of early TT in patients with SDICH. The forest plot displays the regression coefficients (Beta *β*), 95% confidence intervals (CI), and *p*-values for the four variables retained in the final model. A negative *β* value for the GCS indicates an inverse association with the likelihood of early TT, while positive *β* values for white WBC, PLT, and HR suggest positive associations. The dotted vertical line represents the line of null effect (*β* = 0). All four variables were statistically significant independent predictors of early TT (*p* < 0.05).

Multivariate logistic regression analysis using the backward likelihood ratio method identified four independent predictors of early TT in patients with SDICH. As shown in Figure 2, GCS at admission demonstrated a significant inverse association with early TT (*β* = −0.03, 95% CI: −0.04 to −0.03, *p* < 0.001). In contrast, WBC (*β* = 0.01, 95% CI: 0.00–0.02, *p* = 0.003), PLT (*β* = 0.00, 95% CI: 0.00–0.00, *p* = 0.010), and HR at admission (*β* = 0.00, 95% CI: 0.00–0.00, *p* = 0.041) showed significant positive associations with the primary outcome. The forest plot (Figure 2) illustrates the magnitude and direction of these associations, with all four variables remaining significant predictors after adjustment for potential confounders. These parameters were subsequently incorporated into the construction of a dynamic nomogram to predict early TT risk.

### Build the nomogram for early TT

**Figure 3:**
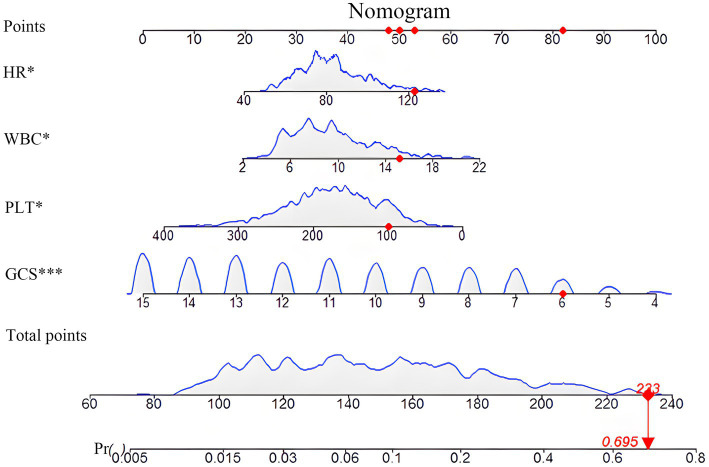
Nomogram for predicting the probability of early tracheotomy. This figure presents the static nomogram developed from the final multivariate logistic regression model for predicting the probability of early tracheotomy in patients with SDICH. To use the nomogram, the value for each predictor (HR, WBC, PLT, and GCS) is located on its corresponding axis, and a vertical line is drawn upward to the “Points” axis to determine the specific score for that variable. The sum of the points for all four predictors is then calculated and located on the “Total Points” axis. Finally, by drawing a vertical line down from the total points to the “Probability of Early Tracheotomy” axis, the individualized predicted probability can be read. The nomogram demonstrates that a lower GCS contributes most significantly to a higher total points value and, consequently, a greater probability of requiring early tracheotomy. The dynamic, interactive version of this nomogram is available online.

An interactive nomogram was developed based on the proposed model (Figure 3). A static version was constructed to facilitate individualized prediction of early TT probability (Figure 3). The nomogram incorporated four variables: GCS score, HR, WBC count, and PLT count. Each variable was assigned a point scale, and the contribution of each predictor was represented by the length of its scale. The GCS had the strongest influence on the total points, followed by PLT, HR, and WBC. To obtain patient-specific risk estimates, the corresponding points for the four variables were summed based on the patients’ admission values. The total score was then projected downward to the probability axis at the bottom of the nomogram to yield the predicted probability of early TT. For example, as shown in Figure 3, a hypothetical patient with specific values for each predictor (represented by red dots) would have a total score of 233 points, corresponding to a predicted probability of 69.5%. An interactive online version of this nomogram is publicly available, enhancing its ease of clinical use.

### The prediction dynamic nomogram model discrimination: ROC curve analysis

**Figure 4:**
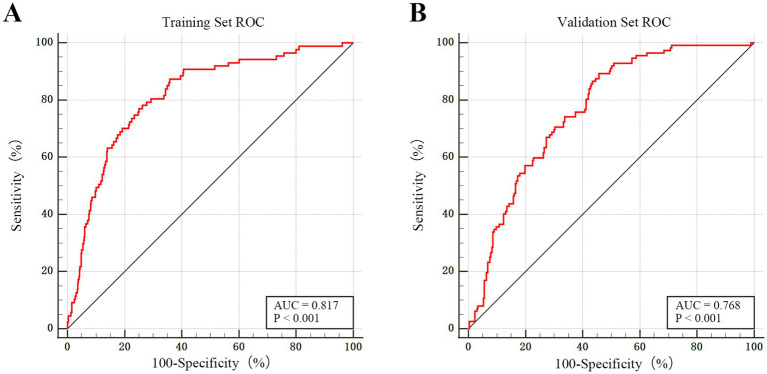
ROC curve in the training and external validation sets. **(A)** ROC curve for the training set (*n* = 667). The model demonstrates excellent discriminatory power, with an AUC of 0.817 (95% CI: 0.785–0.845; *p* < 0.001). **(B)** ROC curve for the external validation set (*n* = 379). The model maintains good predictive performance and generalizability in the independent cohort, with an AUC of 0.768 (95% CI: 0.722–0.809; *p* < 0.001).

The discriminatory performance of the dynamic nomogram was assessed using ROC curve analysis. In the training set, the model showed excellent predictive ability, achieving an AUC of 0.817 (95% CI: 0.785–0.845), sensitivity of 77.01%, specificity of 75%, and a Youden index of 0.520. In the external validation set, the model maintained good discrimination, with an AUC of 0.768 (95% CI: 0.722–0.809), sensitivity of 89.29%, specificity of 54.31%, and a Youden index of 0.400. Comparison of the two ROC curves revealed no statistically significant difference (*p* = 0.147), indicating consistent and robust performance across both cohorts. The optimal probability cutoff values, determined using the Youden index, were >0.139 for the training set and >0.182 for the validation set. These findings confirm the strong capability of the model to distinguish between patients who required early TT and those who did not (see Figure 4).

### The prediction dynamic nomogram model calibration and clinical utility

**Figure 5:**
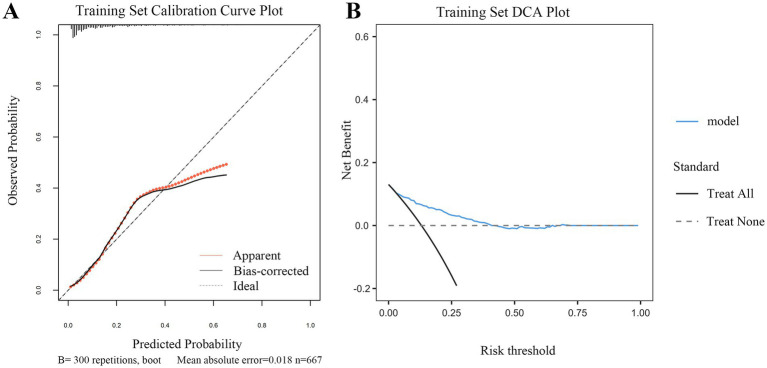
Calibration and clinical utility of the dynamic nomogram in the training set. **(A)** Calibration curve. The plot assesses the agreement between the predicted probabilities of early tracheotomy and the actual observed outcomes. The solid gray line (“Apparent”) represents the model’s performance on the original data. The bias-corrected line (solid dark line), derived from 300 bootstrap repetitions, demonstrates excellent calibration, closely aligning with the ideal line (dashed line) where predictions perfectly match observations. The low mean absolute error of 0.018 indicates high predictive accuracy. **(B)** DCA. The plot evaluates the clinical net benefit of using the nomogram across a range of risk thresholds. The red line (“model”) shows the net benefit provided by the nomogram. The gray line (“Treat All”) represents the strategy of performing early tracheotomy on all patients, and the black line (“Treat None”) represents the strategy of performing it on no one. The DCA demonstrates that using the nomogram for clinical decision-making provides a superior net benefit compared to the “Treat All” or “Treat None” strategies across a wide range of threshold probabilities, confirming its potential clinical utility.

**Figure 6:**
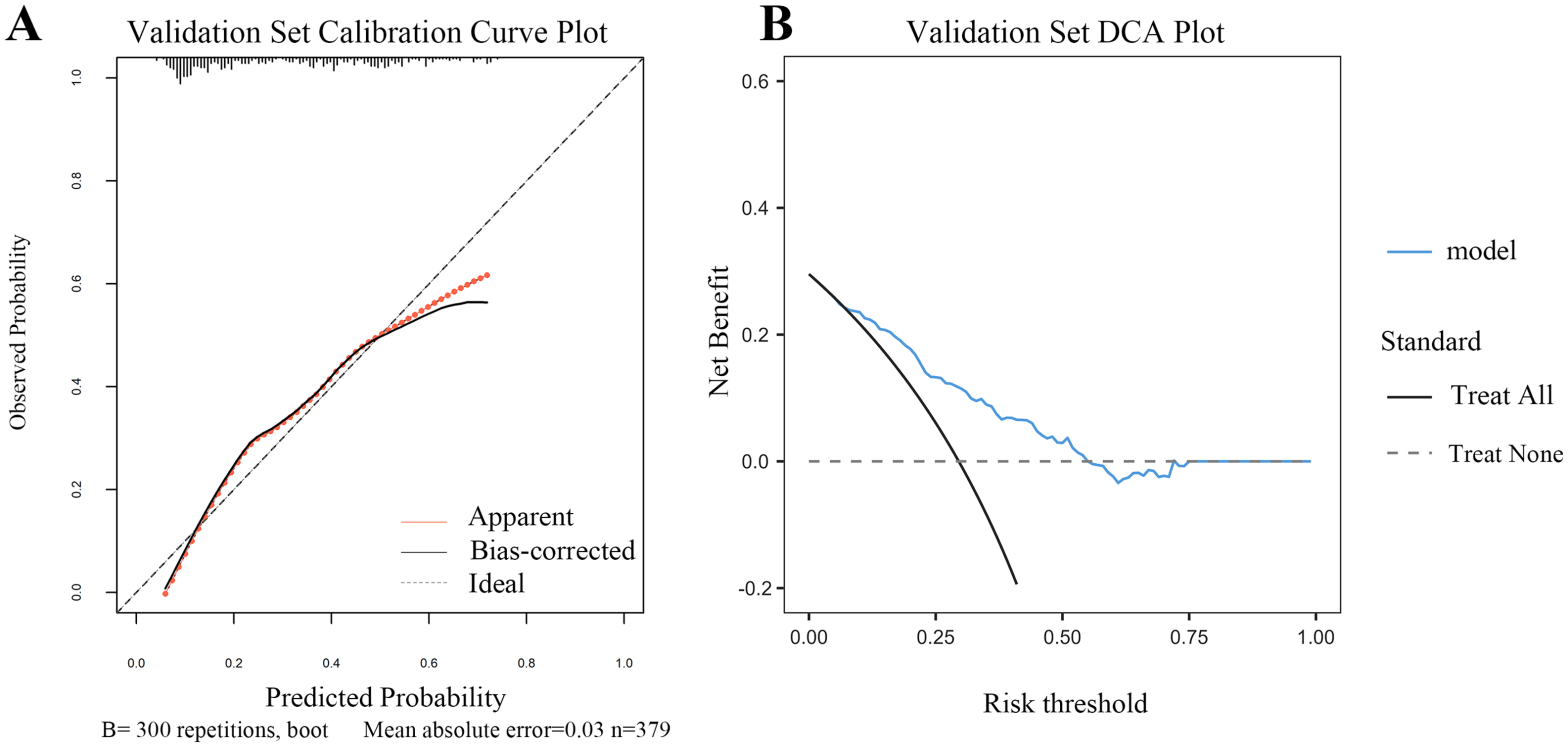
Calibration and clinical utility of the dynamic nomogram in the validation set **(A)** Calibration curve for the validation set. The plot visualizes the agreement between the predicted probabilities from the nomogram and the actual observed frequencies of early tracheotomy in the independent external validation cohort. The solid gray line (“Apparent”) shows the model’s performance. The bias-corrected line (solid dark line), adjusted via 300 bootstrap repetitions, closely follows the ideal line (dashed line), indicating good calibration. The mean absolute error of 0.030 further confirms the model’s satisfactory predictive accuracy in the new patient population. **(B)** Decision curve analysis (DCA) for the validation set. This plot assesses the clinical net benefit of applying the nomogram in the external validation cohort. The red line (“model”) represents the net benefit achieved by using the nomogram to guide decisions. Across a clinically relevant range of risk thresholds, the nomogram strategy provides a higher net benefit than the “Treat All” (gray line) or “Treat None” (black line) strategies. This result validates the potential clinical utility of the model for supporting tracheotomy decisions in diverse clinical settings.

Calibration and clinical utility of the dynamic nomogram were evaluated in both the training and external validation sets. As shown in Figures 5A, 6A, the calibration curves demonstrated excellent agreement between the predicted probabilities of early TT and the observed outcomes. In the training set, the model achieved a *C*-index (ROC area) of 0.817 and a Somers’ *D_xy_* rank correlation of 0.633. It explained a substantial proportion of the variance, with an *R*^2^ value of 0.258. Calibration metrics confirmed strong agreement between predicted and observed outcomes, with a low Brier score of 0.152, indicating high accuracy. The calibration slope was close to the ideal value of 1.000, and the intercept was near zero (0.095), suggesting minimal systematic error. The maximum calibration error (*E*_max_) was 0.205, and the average error (*E*_avg_) was 0.057. This robust calibration performance was replicated in the external validation set, which achieved a *C*-index of 0.768 and a Somers’ *D_xy_* value of 0.535. The model explained a substantial proportion of the variance (*R*^2^ = 0.235) and showed satisfactory calibration, with a Brier score of 0.183. The key calibration parameters performed well, with a slope of 1.000 and an intercept of 0.174, indicating minimal calibration drift. The maximum calibration error (*E*_max_) was 0.115, and the average error (*E*_avg_) was 0.053. These findings confirm that both calibration curves were within excellent ranges.

DCA further confirmed the clinical utility of the model across both cohorts (Figures 5B, 6B). The analysis showed that using the nomogram for clinical decision-making provided a greater net benefit across a wide range of threshold probabilities compared with the strategies of performing TT in all patients or in none. The consistent demonstration of net benefit in both the derivation and validation cohorts highlights the model’s potential as a reliable tool to support individualized clinical decisions regarding early TT in patients with SDICH.

## Discussion

In this study, we developed and validated a dynamic nomogram predictive model for early TT in patients with SDICH. The model integrates key clinical variables, including GCS, WBC, PLT, and HR. These factors are well established in neurocritical care due to their role in predicting respiratory compromise and the need for airway intervention in patients with severe neurological impairment (14, 15). The dynamic nomogram provides an efficient and accessible tool for clinicians, supporting timely decision-making for early TT.

A low GCS score is a hallmark of impaired consciousness and neurological dysfunction in patients with SDICH and often necessitates prolonged mechanical ventilation (16). Reduced GCS scores are closely associated with respiratory complications such as hypoxemia and neurogenic pulmonary edema, both of which can aggravate brain injury and worsen prognosis (17, 18). Early identification of patients with low GCS scores who may benefit from TT is critical for preventing secondary hypoxic damage and improving recovery. In our model, the GCS score was the strongest predictor of the need for early airway intervention, highlighting its essential role (19). An elevated HR is commonly linked to stress responses and pulmonary dysfunction in patients with acute brain injury. Neurogenic pulmonary edema, often caused by sympathetic overactivity, is a recognized complication of severe intracerebral hemorrhage (20, 21). This cycle of hypoxemia and respiratory failure is worsened by increased catecholamine release, which further compromises pulmonary function. In such cases, early TT may break this cycle by reducing airway resistance and improving ventilation. Our inclusion of HR as a predictor aligns with recent studies reporting an association between elevated HR and increased respiratory complications (22). Systemic inflammatory responses, reflected by elevated WBC counts, are common in patients with SDICH and contribute to pulmonary dysfunction (23). A heightened inflammatory state increases the risk of ventilator-associated pneumonia, which further complicates recovery (27). Our findings demonstrate that WBC count is a significant predictor of early TT, reinforcing the relationship between systemic inflammation and the need for timely airway management. In this context, identifying patients requiring TT early could support interventions that reduce pneumonia risk (24, 25). PLT reflects both coagulation and inflammatory responses in patients with SDICH. Low PLT levels indicate coagulopathy or significant blood loss, whereas elevated PLT levels indicate increased inflammatory activity (26). PLT activation in response to intracerebral hemorrhage contributes to local tissue damage and inflammation, increasing the likelihood of respiratory complications. Consistent with previous studies, PLT was found to be a key predictor of early TT in our model (24).

Previous predictive models for early TT, such as the SET and TRACH scores, relied on static clinical and radiological predictors (8, 10). However, these models are limited by their complexity and lack of adaptability to fast-paced clinical settings. Our dynamic nomogram addresses these challenges by providing a real-time, web-based platform that allows clinicians to input patient data and obtain immediate TT predictions. This approach improves the practicality and accuracy of decision-making compared to static models (25).

Recent studies have further highlighted the benefits of early TT. For example, Ding et al. (26) demonstrated that early TT significantly reduced ICU stay and ventilator dependency in patients with brainstem hemorrhage. Similarly, a 2019 meta-analysis by McCredie et al. (15) showed that early TT reduced ventilator-associated complications and was associated with improved outcomes, emphasizing the clinical importance of accurately predicting its necessity. Our findings are consistent with these results, reinforcing the utility of early TT in patients with SDICH and demonstrating the broader clinical applicability of predictive models.

These significant differences between the training and validation sets (Table 2) make the external validation exercise particularly meaningful. The fact that our dynamic nomogram maintained good and statistically significant predictive performance (AUC = 0.768) in a cohort that was sicker and different from the development cohort provides strong preliminary evidence for its robustness and potential transportability. This finding suggests that the core predictive variables (GCS, WBC, PLT, and HR) capture the fundamental physiological derangements associated with the need for early TT and are applicable across varied patient case mixes.

**Table 2:** Comparative analysis of key baseline characteristics between the two sets.

Variable names	Overall	Validation set	Training set	*p*-value
n	1,046	379	667	
Age	58.473 ± 11.276	58.084 ± 11.89	58.694 ± 10.915	0.401
GCS	10.502 ± 3.357	9.726 ± 3.897	10.943 ± 2.919	<0.001
T	36.741 ± 1.149	36.569 ± 1.738	36.839 ± 0.573	<0.001
HR	82.468 ± 15.083	82.211 ± 13.42	82.615 ± 15.958	0.678
SBP	161.551 ± 23.046	168.987 ± 28.999	157.325 ± 17.514	<0.001
DBP	90.475 ± 14.772	93.573 ± 17.019	88.715 ± 13.016	<0.001
Glucose	8.022 ± 2.714	8.626 ± 3.16	7.679 ± 2.359	<0.001
UA	306.246 ± 113.101	333.16 ± 120.375	290.954 ± 105.833	<0.001
K	3.529 ± 0.455	3.543 ± 0.502	3.522 ± 0.427	0.473
Na	138.071 ± 3.896	138.803 ± 4.454	137.655 ± 3.476	<0.001
Ca	2.322 ± 0.167	2.249 ± 0.169	2.364 ± 0.151	<0.001
P	0.906 ± 0.27	0.968 ± 0.309	0.87 ± 0.238	<0.001
Mg	0.848 ± 0.1	0.843 ± 0.11	0.851 ± 0.093	0.247
Albumin	40.058 ± 4.754	38.326 ± 5.494	41.042 ± 3.957	<0.001
WBC	10.205 ± 3.91	11.484 ± 4.34	9.478 ± 3.44	<0.001
Neutrophile	8.352 ± 3.86	9.116 ± 4.367	7.917 ± 3.469	<0.001
Monocyte	0.477 ± 0.248	0.596 ± 0.267	0.409 ± 0.209	<0.001
Hb	146.235 ± 20.047	139.359 ± 18.581	150.142 ± 19.81	<0.001
PLT	196.902 ± 69.157	235.902 ± 67.807	174.741 ± 59.475	<0.001
HV	26.119 ± 20.511	35.438 ± 19.645	20.824 ± 19.067	<0.001
ML	2.377 ± 3.858	5.062 ± 4.031	1.416 ± 3.051	<0.001
Sex (%)				0.440
Male	644 (61.57)	227 (59.89)	417 (62.52)	
Female	402 (38.43)	152 (40.11)	250 (37.48)	
DM (%)				0.800
Yes	133 (12.72)	50 (13.19)	83 (12.44)	
No	913 (87.28)	329 (86.81)	584 (87.56)	
Hypertension (%)				<0.001
Yes	220 (21.03)	146 (38.52)	74 (11.09)	
No	826 (78.97)	233 (61.48)	593 (88.91)	

SBP, systolic blood pressure; DBP, diastolic blood pressure; T, temperature of body; GCS, Glasgow Coma Score; HV (mL), hemorrhage volume; ML (mm), midline line shift; WBC, white blood cell; Hb, hemoglobin; PLT, platelet; UA, uric acid; K, kalium; Na, natrium; Ca, calcium; P, phosphorus; Mg, magnesium. Training set: come from The Second Affiliated Hospital of Fujian Medical University. Validation set: come from The Second Hospital & Clinical Medical School of Lanzhou University.

The dynamic nomogram developed in this study offers several advantages over the traditional models. First, it incorporates readily available clinical variables at admission, facilitating timely and accurate decision-making. Second, its web-based format ensures ease of use across various clinical settings, making the model widely accessible. Third, external validation strengthened its reliability and generalizability. Finally, it is important to emphasize that our model predicts the likelihood of requiring early TT, not the treatment effect of TT itself. The potential clinical utility of this tool lies in its ability to help clinicians identify high-risk patients for closer monitoring and earlier intervention. However, its ultimate effect on patient outcomes requires validation in prospective studies.

### Limitations

While the inclusion of data from two centers enhanced the generalizability of our findings, differences in patient demographics and clinical practices between the centers may restrict broader applicability. Selection bias is another limitation due to the retrospective design of the study: (1) refusal by some families to consent to TT created gaps in the dataset. (2) Not all relevant variables influencing patient outcomes, such as nutritional status, comorbidities, imaging findings, and blood gas analysis results, were included in the model; and (3) clinical judgment regarding the need for TT may vary depending on individual physicians’ experience. In addition, heterogeneity in treatment protocols across the two centers could contribute to variations in TT decision-making. HR, as a model variable and key predictor, is susceptible to external factors such as pain and medication, which may affect its reliability. Moreover, our DCA demonstrated only a moderate benefit, with 45% of patients in the training set and 55% in the validation set benefiting from the model. This limitation may be attributable to the relatively small sample size, particularly in the external validation cohort. Finally, our model did not include direct functional assessments of airway risk factors, such as cough reflex, swallowing function, and tracheal secretions, which are recognized as important predictors of extubation success and TT need. The absence of these variables is a significant limitation, primarily due to inconsistent documentation in retrospective data sources. Future prospective studies that incorporate these functional assessments at admission are necessary to develop more comprehensive and nuanced prediction tools.

## Conclusion

In this study, we developed and validated a dynamic nomogram model for predicting the need for early TT. The model demonstrated that variables such as GCS, HR, WBC count, and PLT can reliably predict the necessity of early TT in patients with SDICH. However, further prospective, multicenter studies are required to confirm and strengthen our findings.

## Data Availability

The raw data supporting the conclusions of this article will be made available by the authors, without undue reservation.

## Supplementary material

SUPPLEMENTARY FIGURE 1 RCS curve.
