# Supplementary figures and images for: Dynamic nomogram for predicting early tracheotomy in patients diagnosed with supratentorial deep seated intracranial hemorrhage
Source: Front Neurol. 2025 Nov 5;16:1670672. doi: 10.3389/fneur.2025.1670672 (PMC12627028; doi:10.3389/fneur.2025.1670672)

RCS Prediction Plot

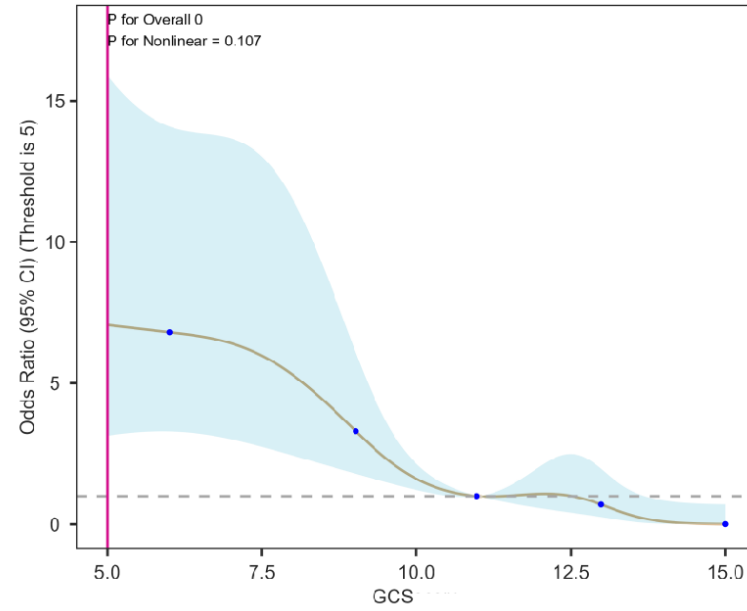

RCS Prediction Plot

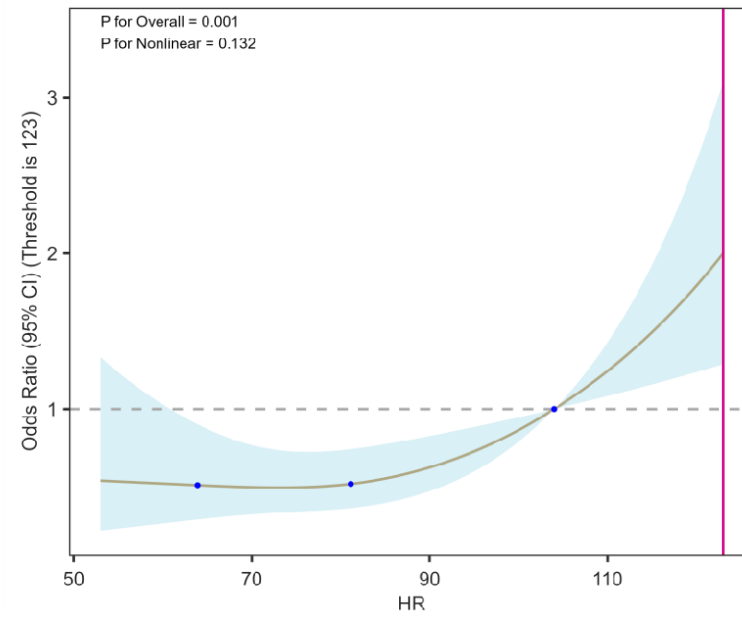

RCS Prediction Plot

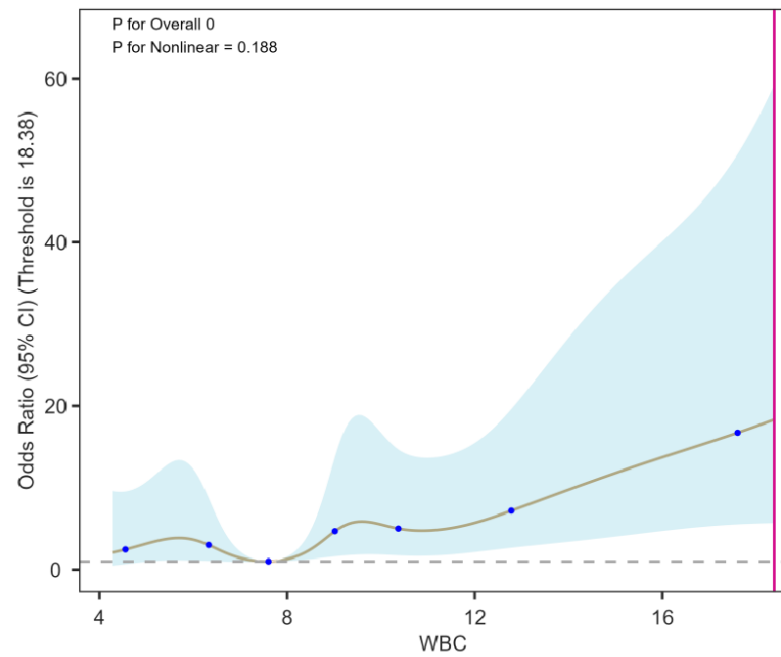

RCS Prediction Plot

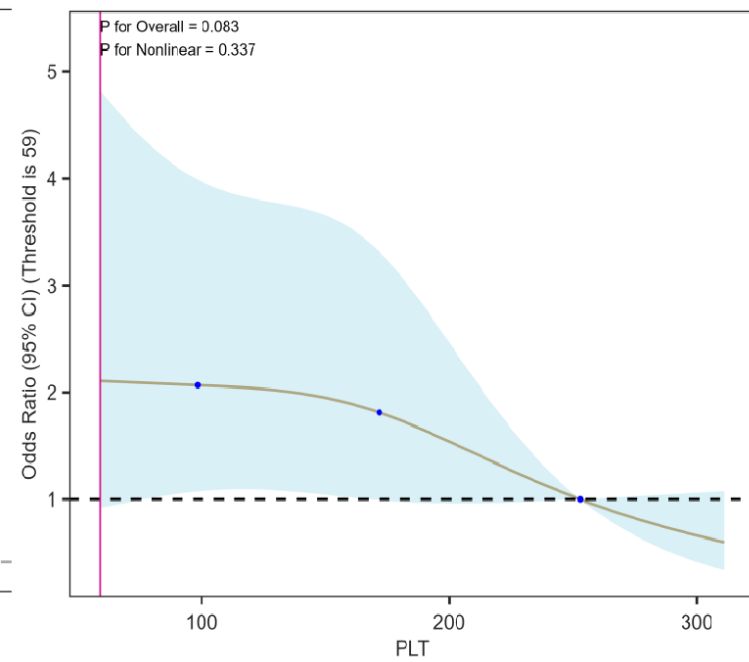

Supplement: SUPPLEMENTARY FIGURE 1 — RCS curve. [file Data_Sheet_1.PDF]
