# Supplementary material for: Dynamic nomogram for predicting early tracheotomy in patients diagnosed with supratentorial deep seated intracranial hemorrhage
Source: Front Neurol. 2025 Nov 5;16:1670672. doi: 10.3389/fneur.2025.1670672 (PMC12627028; doi:10.3389/fneur.2025.1670672)
Supplement: Supplementary file 2 [file Table_1.DOCX]

**Supplementary table 1 VIF analysis**

| **Variable Name** | **GVIF** | **Df** | **VIF** |
| --- | --- | --- | --- |
| **GCS** | 1.177 | 11 | 1.007 |
| **PLT** | 1.126 | 1 | 1.061 |
| **HR** | 1.020 | 1 | 1.010 |
| **WBC** | 1.221 | 1 | 1.105 |
