# Supplementary material for: Dynamic nomogram for predicting early tracheotomy in patients diagnosed with supratentorial deep seated intracranial hemorrhage
Source: Front Neurol. 2025 Nov 5;16:1670672. doi: 10.3389/fneur.2025.1670672 (PMC12627028; doi:10.3389/fneur.2025.1670672)
Supplement: Supplementary file 3 [file Table_2.DOCX]

**Supplementary table 2 variables nonlinear relationships**

| **Row Name** | **Chi_Square** | **Df** | **P value** |
| --- | --- | --- | --- |
| **GCS-Nonlinear** | 6.091 | 3 | 0.107 |
| **HR-Nonlinear** | 2.271 | 1 | 0.132 |
| **WBC-Nonlinear** | 7.47 | 5 | 0.188 |
| **PLT-Nonlinear** | 0.922 | 2 | 0.337 |
